# The Influence of Menopause and Inflammation on Redox Status and Bone Mineral Density in Patients with Rheumatoid Arthritis

**DOI:** 10.1155/2021/9458587

**Published:** 2021-01-07

**Authors:** Aleksandra Stojanovic, Mirjana Veselinovic, Nevena Draginic, Marina Rankovic, Marijana Andjic, Jovana Bradic, Sergey Bolevich, Aleksandra Antovic, Vladimir Jakovljevic

**Affiliations:** ^1^University of Kragujevac, Faculty of Medical Sciences, Department of Pharmacy, Kragujevac, Serbia, Svetozara Markovica 69, 34000 Kragujevac, Serbia; ^2^University of Kragujevac, Faculty of Medical Sciences, Department of Internal Medicine, Kragujevac, Serbia, Svetozara Markovica 69, 34000 Kragujevac, Serbia; ^3^I.M. Sechenov First Moscow State Medical University, Department of Human Pathology, Trubetskaya str. 8, 119991 Moscow, Russia; ^4^Department of Medicine, Rheumatology Unit, Karolinska Institutet, Stockholm, Sweden; ^5^Academic Specialist Center, Center for Rheumatology, Stockholm Health Services, Stockholm, Sweden; ^6^University of Kragujevac, Faculty of Medical Sciences, Department of Physiology, Svetozara Markovica 69, 34000 Kragujevac, Serbia

## Abstract

Although oxidative stress is considered to be one of the key pathogenic factors in rheumatoid arthritis (RA), there is insufficient knowledge regarding the impact of menopause on redox status in this population. Thus, this study is aimed at assessing the influence of menopause within healthy women and within RA patients as well as the impact of RA in premenopausal and postmenopausal women on redox status, with special reference to bone mineral density (BMD). A total of 90 women were included in the study, 42 with RA and 48 age-matched healthy controls. They were divided into subgroups according to the presence of menopause. Following oxidative stress parameters were measured spectrophotometrically: index of lipid peroxidation (measured as TBARS), nitrites (NO_2_^−^), superoxide anion radical (O_2_^−^), hydrogen peroxide (H_2_O_2_), superoxide dismutase (SOD), catalase (CAT), and reduced glutathione (GSH). BMD was assessed by using a dual-energy X-ray absorptiometry scanner. Comorbidities and drug history were recorded. The levels of H_2_O_2_ and TBARS were elevated in patients with RA, while NO_2_^−^ and O_2_^−^ increased in healthy women, both in premenopausal and postmenopausal groups. SOD activity decreased in postmenopausal RA patients. BMD was reduced in postmenopausal RA women. There was a correlation between NO_2_^−^ and O_2_^−^ with Health Assessment Questionnaire (HAQ) index in RA patients. Given that postmenopausal state was associated with elevated oxidative stress within healthy women and that menopausal state did not affect redox homeostasis within RA patients, but the redox homeostasis was altered in both RA groups compared to healthy women, it can be presumed that impaired redox status in RA occurred due to presence of the disease, irrespective of age. Moreover, menopause attenuates BMD reduction in women with RA. These results may indicate the need for therapeutic use of antioxidants in the form of supplements in women with RA, regardless of age.

## 1. Introduction

Rheumatoid arthritis (RA) is a chronic, inflammatory, and autoimmune disease, causing synovitis and destructive arthritis with progressive cartilage and joint damage. These local signs are accompanied by out-of-joint manifestations including a strong systemic inflammatory response with accelerated development of atherosclerosis, disabilities, shortened life expectancy, and as such represent a significant burden for both individuals and society [1–5]. RA affects almost 1% of the world's total population, with a ratio of 6 : 1 in the favor of women in younger population and 2 : 1 in the 55-64-year-old population [5, 6]. In people over 75 years of age, the incidence becomes either equal or higher in male population. The most common onset of the illness is in the forties [7]. Female subjects experience a higher activity of RA in comparison with male patients [8]. Patients with RA have an increased rate of morbidity and a high prevalence of cardiovascular disease (CVD), hypercoagulability, and consequently increased risk of developing fatal cardiovascular events such as acute myocardial infarction, stroke, and heart failure [9–11].

The key pathogenic mechanisms involved in almost all autoimmune diseases are oxidative stress and inflammation. Under physiological conditions, small quantities of reactive oxygen species (ROS) are beneficial for cellular and mitochondrial signaling and function [12]. However, higher ROS production amounts lead to oxidative stress that can mediate cell and tissue damage and secondary inflammation [12]. Oxidative stress represents an imbalance between prooxidative and antioxidant parameters in favor of prooxidants [13]. Oxidative stress and chronic inflammation cross-promote each other and contribute to CVD progression even in the subacute phase of the disease [14, 15].

Recently, oxidative stress generated as a result of reduced antioxidant protection has become a major finding in patients with RA [12, 16]. What is more, oxidative stress is positively correlated with inflammation and accelerated joint destruction in RA patients [17–19]. Although it becomes easy to admit the potential cross talk between oxidative stress and RA, few studies were devoted to comprehend the in-depth mechanisms through which redox imbalance contributes to the establishment of a proinflammatory milieu observed in RA and vice versa [20, 21].

A controversial association between the onset of menopause and rheumatic disease development has been reported [5]. A recent study has shown that early menopause (before 45 years of age) is associated with an increased risk of developing postmenopausal (PostM) RA [22], while another suggests that early menopause may be associated with a milder course of the disease [23]. In addition, menopause increases the risk of osteoporosis and fracture occurrence [24]. Furthermore, it is known that oxidative stress plays an important role in the aging process, resulting in increased free radical's concentrations, which is not followed by an equal increase in antioxidant defence mechanisms [25]. However, there is still a knowledge gap regarding the impact of menopause on redox status and bone mineral density in patients with RA.

Taking into consideration our previous findings, impaired oxidative stress in RA patients [16] and significant difference in hemostatic potential between healthy women and RA patients analysed in relation to menopausal state [11], we assumed that potential differences in redox status between RA patients and healthy controls also might be investigated in the aspect of menopause. We wanted to examine whether there is any difference in redox state within premenopausal and within postmenopausal RA patients and weather there is any influence of menopause on redox state. Another novelty reflects in assessment of the changes in bone mineral density between RA and healthy controls, concerning menopause. Therefore, this study is aimed at assessing the influence of menopause within healthy women and within RA patients as well as the impact of the RA in premenopausal and postmenopausal women on oxidative stress status and comparing to the healthy women of the same age, with special reference to bone mineral density. This is a novel study providing a complete picture about the influence of menopause on redox status as well as impact of RA on redox alteration at different ages, in pre- and postmenopausal patients.

## 2. Patients and Methods

### 2.1. Patients

A total of 42 women with diagnosed RA referred to the outpatient clinic, Department of Rheumatology, Clinical Centre Kragujevac, Serbia, were included in the study (mean age 54.8 ± 9.1). The diagnosis of RA was established according to the classification criteria of the American College of Rheumatology (ACR)/European League Against Rheumatism (EULAR) 2010 [26]. All patients were treated with the standard treatment protocol methotrexate (15-25 mg per week) and prednisolone (≤10 mg per day). The exclusion criteria included a history of diabetes, malignancy, inflammatory disease except rheumatoid arthritis, liver-, or renal insufficiency, and previous hospitalization due to cerebrovascular, cardiovascular disorders, and venous thromboembolism. RA assessments included a detailed medical history, the presence of extra-articular disease manifestation, current disease activity assessed by the 28-point disease activity score (DAS28) [27], and physical function using the Health Assessment Questionnaire (HAQ) [28]. All current medications were recorded. Additionally, at the time of blood sampling, patients were free of any medication or supplemental antioxidant therapy known to influence redox status. Moreover, the blood sampling was obtained one day before standard methotrexate therapy, and patients were free of NSAIDs at least three days before blood sampling, with the optional use of topical NSAIDs in the case of need for analgesic medication.

### 2.2. Healthy Controls

The total of 48 age-matched female subjects (mean age 54.1 ± 6.2) were included in the study as healthy controls. The exclusion criteria were diabetes, malignancy, and inflammatory disease including rheumatoid arthritis, liver-, or renal insufficiency. As well as in patients' group, the controls were free of any medication known to influence redox status.

All women included in the study were not taking oral contraceptives or hormone replacement treatment. All hormone factors were self-reported by the participants. Postmenopausal status was defined as cessation of menses for 12 months [29].

Women in both groups were divided into the following four different subgroups according to menopause: (1) premenopausal (PreM) controls (*n* = 14), (2) postmenopausal (PostM) controls (*n* = 34), (3) PreM RA patients (*n* = 11), and (4) PostM RA patients (*n* = 31). The oldest menstruating control was 53 years old, and the youngest menopausal woman with RA was 45 years old.

The written informed consent was obtained from all participants, and the study protocol was approved by the Ethics Committee of the Clinical Center Kragujevac prior to the onset of the study. The investigation was conducted in accordance with the principles outlined in the Declaration of Helsinki and principles of Good Clinical Practice (GCP).

### 2.3. Blood Sampling

Blood sampling was obtained in the same manner for all patients at the Internal Clinic, Department of Rheumatology, Clinical Center Kragujevac, Serbia. Venous blood from all participants was collected between 8 and 10 am, following at least a 10-hour fasting period, in a quiet, air-conditioned, and temperature-controlled room (22–24°C). Blood was collected in Vacutainer tubes containing 0.129 M sodium citrate (BD Vacutainer Blood Collection System) using 21-gauge polyethylene catheter for taking blood samples (BD Vacutainer needles). Blood was centrifuged to separate plasma and red blood cells (RBCs) and stored at −20°C.

### 2.4. Redox Status

Redox status was evaluated spectrophotometrically by measuring the index of lipid peroxidation (measured as TBARS), nitrites (NO_2_^−^), superoxide anion radical (O_2_^−^), and hydrogen peroxide (H_2_O_2_) in plasma. Activities of corresponding antioxidative enzymes superoxide dismutase (SOD), catalase (CAT), and the concentration of reduced glutathione (GSH) were measured in erythrocytes in the same manner [16].

#### 2.4.1. Index of Lipid Peroxidation (Thiobarbituric Acid Reactive Substances)

The degree of lipid peroxidation in plasma was assessed by TBARS measuring using 0.4 ml 1% thiobarbituric acid (TBA) in 0.05 NaOH mixed with 0.8 ml of plasma, incubated at 100°C for 15 min, and measured at 530 nm. Distilled water was used as a blank probe. TBA extract was obtained by combining 0.8 ml plasma and 0.4 ml TCA (trichloroacetic acid). Thereafter, samples were put on ice for 10 min and centrifuged for 15 min at 6000 rpm [16, 30].

#### 2.4.2. Nitrite Determination

NO decomposes rapidly to form stable metabolite nitrite/nitrate products. The method for detection of the plasma nitrite levels is based on the Griess reaction. Nitrites (NO_2_^−^) were determined as an index of NO production with Griess reagent (forms purple diazocomplex) [16, 31]. The total of 0.1 ml 3 N PCA (perchloric acid), 0.4 ml 20 mM EDTA (ethylenediaminetetraacetic acid), and 0.2 ml plasma were put on ice for 15 min, then centrifuged for 15 min at 6000 rpm. After pouring off the supernatant, 220 *μ*l K_2_CO_3_ was added. Nitrites were measured at 550 nm. Distilled water was used as a blank probe.

#### 2.4.3. Superoxide Anion Determination

The level of O_2_^−^ was measured using Nitro Blue Tetrazolium (NBT) reaction in TRIS-buffer with plasma and read at 550 nm. Distilled water was used as a blank probe [16, 32].

#### 2.4.4. Hydrogen Peroxide Determination

Determination of H_2_O_2_ concentration is based on oxidation of phenol red using H_2_O_2_, in the reaction catalyzed by enzyme peroxidase from horseradish (POD) [16, 33]. The total of 200 *μ*l sample with 800 μl PRS (phenol red solution) and 10 μl POD were combined (1 : 20) and measured at 610 nm.

### 2.5. Determination of Catalase, Superoxide Dismutase, and Reduced Glutathione

Isolated RBCs were washed three times with 3 volumes ice-cold 0.9 mmol/l NaCl and hemolysate containing about 50 g Hb/l, prepared according to McCord and Fridovich [16, 34], and were used for the CAT activity determination. Determination of CAT activity was performed according to Beutler [16, 35]. Lysates were diluted with distilled water (1 : 7 v/v) and treated with chloroform–ethanol (0.6 : 1 v/v) to remove hemoglobin. Then, 50 *μ*l CAT buffer, 100 μl sample, and 1 ml 10 mM H_2_O_2_ were added to the samples. Detection was performed at 360 nm. Distilled water was used as a blank probe. Determination of SOD activity is based on epinephrine method of Misra and Fridovich [16, 36]. A 100 *μ*l lysate and 1 ml carbonate buffer were mixed, and then, epinephrine in a volume of 100 μl was added. Detection was performed at 470 nm. This method belongs to the “negative” type group of methods, since it monitors a decrease of autoxidation speed in alkaline medium, which is dependent of O_2_^−^. The level of GSH concentration was determined based on GSH oxidation with 5.5-dithiobis-6.2-nitrobenzoic acid, using Beutler method [16, 37]. Measurement of the absorbance is carried out at a wavelength of maximum absorption of 420 nm.

### 2.6. Bone Mineral Density (BMD) Assessment by Dual-Energy X-Ray Absorptiometry (DXA)

The participants had been referred for evaluation of bone mineral density (BMD) by their general practitioners, and the examinations were performed using a DXA (dual-energy X-ray absorptiometry) scanner. DXA of the lumbar spine and proximal femur was performed using standard techniques on a Hologic Horizon DXA. The cutoff for osteoporosis was set as a *T* score ≤ −2.5 at any measured location as per common practice. Osteopenia was defined as a *T* score between −1 and −2.5, and normal BMD was defined as a *T* score ≥ −1 using the lowest reported *T* score. In nonmenopausal women, a *Z* score was followed, with the same cutoff values as for the *T* score [38].

### 2.7. Routine Laboratory Analysis

Laboratory analyses of CRP (Turbidimetric method, Beckman Coulter AU680 analyzer), ESR (Westergren method, Vacuette ESR analyzer), fibrinogen concentration (Clauss method, ACL TOP analyzer by Instrumentation Laboratory), lipid profile (cholesterol, triglycerides, HDL, and LDL, all by Beckman Coulter AU680 analyzer), rheumatoid factor (RF) (turbidimetric method, Beckman Coulter AU680 analyzer), and anti-citrullinated protein antibodies (ACPA) (Roche electrochemiluminescence immunoassay, Cobas e411 analyzer) were performed in the Central Laboratory of the Clinical Center Kragujevac [11].

### 2.8. Statistical Analysis

All data were analysed using SPSS 20.0 (IBM Corp. Released 2011) and GraphPad Prism 5 (Version for Windows, GraphPad Software, La Jolla California, USA). The results are expressed as means ± standard deviation of the mean (SD), median, or as percentages depending on the data type. Distribution of the data was checked by the Shapiro–Wilk test. Independent samples *t*-tests (parametric) and Mann–Whitney *U*-tests (nonparametric) were used to assess the difference in estimated variables between the groups. Correlation between variables was examined by using Spearman correlation analysis depending on the data type and distribution. A *p* value <0.05 was regarded as statistically significant.

## 3. Results

### 3.1. Characteristics of the Study Population and Disease Activity

The mean disease duration in patients was 12.8 ± 8.0 years, and disease activity was medium to high (DAS28 was 3.8 ± 1.1); only 9 patients (20%) were in remission (DAS28 < 2.6); HAQ score was 1.26 ± 0.24, at the moment of blood sampling [11, 16]. Detailed demographic characteristics and disease activity in the followed subgroups are presented in our previous work [11, 16].

### 3.2. Laboratory Parameters in the Study Population

When it comes to nonspecific parameters of inflammation, the patients with RA had higher CRP (11.5 ± 6.4 mg/L vs. 2.5 ± 2.4 mg/L, *p* = 0.0001), ESR (22.9 ± 13.5 mm/h vs. 13.3 ± 8.8 mm/h, *p* = 0.0001), and fibrinogen levels (3.5 ± 0.7 g/L vs. 3.2 ± 0.5 g/L, *p* = 0.031) compared to the controls [16]. The levels of CRP, ESR, and fibrinogen were lower in PreM controls compared to PostM women in the control group, but the difference ceased to exist in the RA group, with the highest values in the PreM RA group (Table 1) [11].

### 3.3. Major Comorbidities of the Study Population

Table 2 shows detailed prevalence of major comorbidities at baseline in the followed groups. Hypertension (37.5% vs. 42.8%) and arrhythmia (8.3% vs. 11.9%) can be distinguished as the two comorbidities with the highest prevalence in both control and RA groups. Other comorbidities present in the study were hyperthyroidism (4.2% vs. 2.4%), Hashimoto thyroiditis (4.8% vs. 7.1%), hypercholesterolemia (4.2% vs. 4.8%), gallbladder calculosis (4.8% in the RA group), bronchial asthma (4.8% in the RA group), peptic ulcer (6.3% vs. 7.1%), gout (2.1% in the control group), glaucoma (2.1% in the control group), and migraine (2.1% in the control group).

### 3.4. Medical History of the Study Population

The mostly used drugs in both control and RA groups were antihypertensives, ACE inhibitors (37.5% vs. 33.3%) and beta-blockers (18.7% vs. 23.8%). Other used drugs were Ca-antagonists (4.2% vs. 7.1%), diuretics (2.1% in the control group), statins (4.2% vs. 4.8%), phlebotonics (2.1% vs. 4.8%), thionamide (4.2% vs. 2.4%), thyroid hormones (4.2% vs. 14.3%), proton pump inhibitors (6.3% vs. 19.0%), bronchodilator (4.8% in RA group), H_1_-antagonists (4.2% in control group), xanthine oxidase inhibitor (2.1% in control group), prostaglandin analogues (2.1% in control group), and ergot alkaloids (2.1% in control group).  Table 3 shows detailed drug usage in the followed groups.

### 3.5. Redox Status

**Figure 1:**
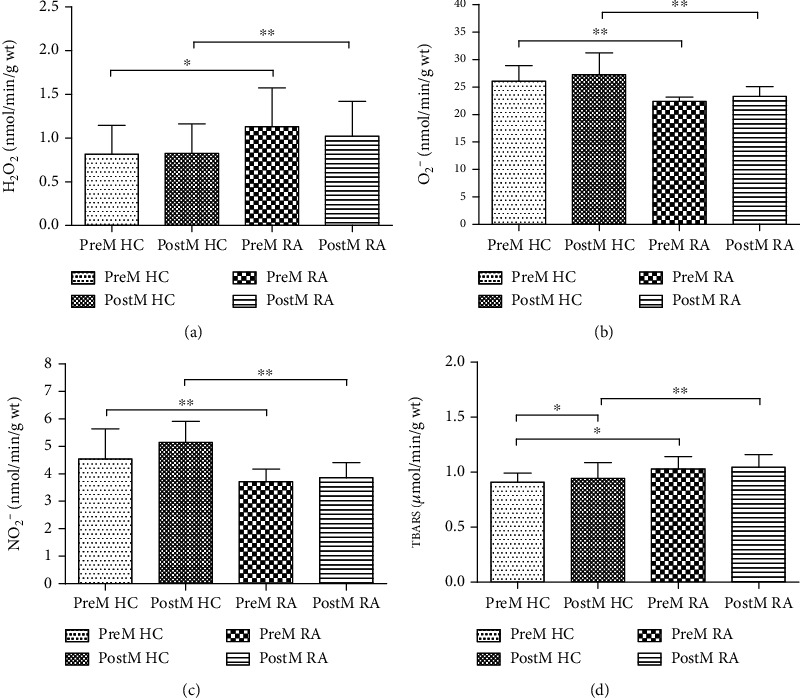
The values of prooxidative parameters in healthy controls and patients with RA in relation to menopause status. (a) Hydrogen peroxide (H_2_O_2_); (b) superoxide anion radical (O_2_^−^); (c) nitrites (NO_2_^−^); (d) index of lipid peroxidation (measured as TBARS). HC: healthy control; RA: rheumatoid arthritis; PreM: premenopausal; PostM: postmenopausal. ^∗^*p* < 0.05; ^∗∗^*p* < 0.01. Data are presented as a mean ± SD.

**Figure 2:**
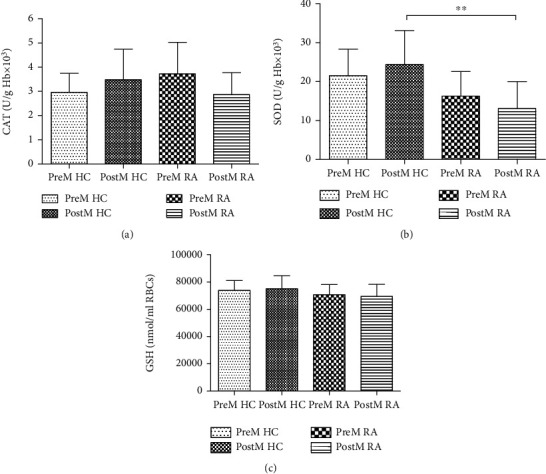
The activity of antioxidative defence system in healthy controls and patients with RA in relation to menopause status. (a) Catalase (CAT); (b) superoxide dismutase (SOD); (c) reduced glutathione (GSH). HC: healthy control; RA: rheumatoid arthritis; PreM: premenopausal; PostM: postmenopausal. ^∗∗^*p* < 0.01. Data are presented as a mean ± SD.

The dynamics of prooxidative parameters (H_2_O_2_, O_2_^−^, NO_2_^−^, and TBARS) in healthy control and patients with RA are shown in Figure 1, while activities of the antioxidative enzymes (SOD, CAT, and GSH) are shown in Figure 2.

#### 3.5.1. Values of Prooxidative Parameters

The highest H_2_O_2_ concentration was noticed in PreM RA group. Significantly higher H_2_O_2_ values were found in PreM RA group compared to PreM HC. Moreover, significantly higher H_2_O_2_ values were found in PostM RA group compared to PostM HC (Figure 1(a)). O_2_^−^ and NO_2_^−^ show a similar trend. Significantly higher O_2_^−^ and NO_2_^−^ values were found in PreM HC group compared to PreM RA as well as higher O_2_^−^ and NO_2_^−^ values in PostM HC group compared to PostM RA (Figures 1(b) and 1(c)). The index of lipid peroxidation measured as TBARS was the highest in PostM RA group. There was significant increased TBARS level in PreM RA group in comparison to PreM HC and in PostM RA groups in comparison to PostM HC. Furthermore, significant difference was noticed between Pre- and PostM HC, with higher TBARS values in PostM healthy women (Figure 1(d)).

#### 3.5.2. Activity of Antioxidant Parameters

The CAT activity and GSH values were not statistically different between the observed groups (Figures 2(a) and 2(c)). SOD activity was significantly decreased in PostM RA in comparison to PostM HC group. The highest SOD activity was noticed in PostM HC (Figure 2(b)).

### 3.6. BMD according to DXA

In PreM RA group, twice less women were found with normal BMD compared to PreM HC (36.4% vs. 71.4%). In PostM RA group, five-time less women were found with normal BMD compared to PostM HC (9.7% vs. 44.1%). Osteopenia was found in 63.6% PreM RA patients compared to 28.6% in PreM HC as well as in 71.0% PostM RA patients compared to 41.2% in PostM HC. In both, the ratio is 2 : 1 in favor of RA patients with osteopenia compared to HC. Osteoporosis was not found in both, PreM RA and PreM HC patients, while osteoporosis was diagnosed in 19.3% of PostM RA patients compared to 14.7% in PostM healthy women (Table 4).

### 3.7. Correlations of Parameters of Redox Status with Parameters of Disease

**Figure 3:**
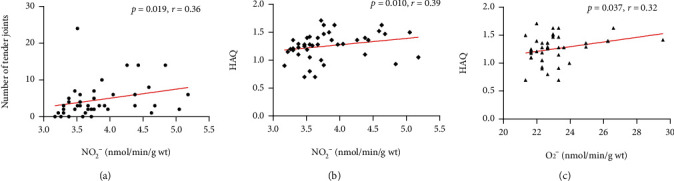
Correlation of ROS with laboratory and clinical parameters. NO_2_^−^: nitrites O2-: superoxide anion radical; HAQ: Health Assessment Questionnaire.

Values of NO_2_^−^ positively correlated with number of tender joints (*p* < 0.05), while the values of NO_2_^−^ and O_2_^−^ positively correlated with HAQ (*p* < 0.05, respectively). Correlations of prooxidative parameters and antioxidative enzyme activity with laboratory analysis have not been found. Significant correlation of the examined parameters is presented in Figure 3.

## 4. Discussion

In our study, we aimed to examine the impact of menopause and RA within premenopausal and within postmenopausal patients on systemic redox status, as well as their connection with BMD. We have explored oxidative stress by assessing both prooxidative parameters and markers of the antioxidative defence mechanism. Our previous work with the main focus on oxidative stress has shown impairment in redox status in RA patients [16]. Moreover, another study noticed the influence of RA as autoimmune inflammatory disease on hemostatic disturbances in the groups according to menopausal state. There was no difference between women with RA and controls in hemostatic potential; however, a significant difference was observed in subgroups analysed in relation to menopausal state. These findings presumably indicated that the inflammatory burden in women with RA activates hemostasis and closes the difference between pre- and postmenopausal RA patients regarding hemostatic activation [11]. Taking into consideration of the aforementioned findings, we assumed that potential differences in redox status between RA patients and healthy controls might be investigated in relation to the occurrence of menopause, within pre- and postmenopausal healthy women and RA patients. The major advantage of our study is the extensive investigation of oxidative stress parameters and BMD in a homogenous group of RA patients concerning menopause in comparison with strictly matched control subjects.

Oxidative stress enhances inflammation in chronic conditions, such as RA and its complications [12]. Enhanced oxidative stress has been implicated in the pathogenesis and progression of articular and cardiovascular damages by promoting protein redox-dependent modifications and cytokine production that further increase prooxidative agents in a vicious circle [14]. Our previous work confirmed that impaired redox status was observed in RA patients. It has been shown that prooxidative parameters were increased in RA patients, while antioxidative system of defence was disturbed [16].

The levels of H_2_O_2_ and TBARS were shown to be elevated in patients with RA compared to healthy women [16, 39]. In our study, the most prominent influence of menopause on TBARS level was noticed in healthy women, which suggests that menopause promotes lipid peroxidation and alters lipid integrity in healthy conditions. This is in accordance with previous studies, suggesting higher markers of lipid peroxidation and carbonyls products as well as low antioxidant defence in PostM women, indicating that these women were exposed to oxidative stress [40–42]. Hormonal status associated with menopause most likely leads to increased oxidative process. Estrogens may have an antioxidant effect due to their capability to protect against 8-hydroxylation of guanine DNA basis. On the other hand, low concentrations of estrogen in conditions such as menopause together with the fact that catechol estrogene's metabolites have a prooxidative effect, leading to oxidation of bases, are the major causes of oxidative stress in this specific population [43]. A significant increase in index of lipid peroxidation was observed in healthy women when analysed in relation to menopausal state. This difference was not found in RA patients according to menopause. These findings presumably may indicate that the inflammatory burden in both pre- and postmenopausal women with RA disturbs the balance of redox status and equals the values between pre- and postmenopausal RA patients, whereby within healthy subgroups, this difference exists.

Interestingly, in our study, the values of nitrites were significantly higher in healthy women compared to RA patients, both in premenopausal and postmenopausal women. Given the fact that the half-life of NO is around 0-10 seconds, direct NO quantification is very difficult so it was measured as stable anions–nitrites [44]. A similar trend was noticed for O_2_^−^ concentration. Previous study has shown higher values of NO_2_^−^ in healthy women compared to RA patients [16]. According to literature data, a profound decrease of NO level in RA patients might be due to the hyperactivity and uncoupling of the NO forming enzyme—nitric oxide synthase (NOS). Activation of inducible NOS form (iNOS) leads to endothelial dysfunction by depleting the bioavailability of tetrahydrobiopterin (BH4) from endothelial NOS (eNOS), resulting in uncoupled eNOS and consequently production of O_2_^−^, rather than NO. Moreover, increased level of myeloperoxidase (MPO) from neutrophils leads to a high consumption of NO. In chronic inflammatory diseases, including RA, inflammation could both reduce NO production and rapid NO destruction via eNOS uncoupling with subsequent ROS production, with a net NO reduction as described for RA patients [45]. In addition to systemic changes, the increase in ROS/RNS is most commonly found locally in affected joints, especially O_2_^−^, H_2_O_2_, OH, NO, ONOO^−^, and HOCl levels [17]. Important sources of NO are joint chondrocytes and synovial fibroblasts. Inflammatory cells such as neutrophils, lymphocytes, mast cells, and macrophages are also important sources of NO [46]. Freely available NO and O_2_^−^ react with each other to form the highly reactive peroxynitrite (ONOO-) [47]. The oxidant potential of ONOO- is greater than that of O_2_^−^ or H_2_O_2_, due to inability to escape from the cells because of its high molecular weight [48]. The local increase in highly reactive radical species is supported by the finding of elevated levels of peroxynitrite and nitrothyrosine in the synovial fluid of patients with RA [49]. Regarding, we have found correlation between NO_2_^−^ and O_2_^−^ and physical function in patients with RA measured by using the HAQ and between NO_2_^−^ and number of tender joints. This correlation may be related to the aforementioned locally increased ROS/RNS with a possible direct impact on the joints.

A significant drop in SOD activity was noticed in older postmenopausal RA patients compared to healthy women of the same age, thus confirming a decreased antioxidant defence in postmenopausal RA patients. As the major antioxidant enzyme, SOD is presented in the vascular wall. Its role is to neutralize O_2_^−^, a proinflammatory ROS, and convert it to hydrogen peroxide, a more diffusible ROS [50, 51]. Menopause did not influence the SOD activity within the control and within the RA group. In line with our results, previous study showed unchanged SOD activity in postmenopausal compared to premenopausal [41]. RA strongly decreased the SOD activity in postmenopausal patients compared to postmenopausal healthy women while menopause mostly equalised values within control and within RA group. The SOD activity was not affected in the premenopausal RA women, compared to healthy control. Decreased level of SOD enzyme in PostM RA patients could be due to compensatory response to elevated oxidative stress induced by low estrogen concentration, as SOD is an inducible enzyme [43]. In line with our results, the previous study reported a significant decreased SOD level in RA subjects [16, 44]. Nonetheless, a lower SOD activity was observed during longer disease duration [52].

In our study, we did not find a significant influence of menopause on the CAT activity and GSH level, both in healthy or in RA diagnosed women. Moreover, CAT activity and GSH level were not changed in premenopausal or postmenopausal women with established RA. In line with our research, the previous study reported unchanged CAT and glutathione peroxidase activities in RA patients compared to healthy control [39, 53], suggesting these molecules may not play a major role in inflammation in RA [53].

We also aimed to determine BMD alterations related to menopause and RA. In patients with RA, osteoporosis is considered an extra-articular disease manifestation. The prevalence of the systemic bone loss in RA patients is 56% in postmenopausal women and 18% in premenopausal and almost twice as low as in healthy women at the same age [54, 55]. *T* score is widely used to estimate risk of fractures, with values between -1 and -2.5 being associated with a 17.6% increased risk for fracture while *T* score higher than -2.5 increases the risk up to 46.3%. The normal bone mineral density was observed in 71.4% of healthy PreM women and expectedly in smaller percentage (44.1%) in PostM healthy women. The lowest number of diagnoses of osteopenia was in healthy women before menopause (28.6%), while the highest percentage was in the population of women with RA in menopause (71%). Osteoporosis was not observed in PreM women in both study groups, so it can be presumed that RA did not affect BMD in premenopausal women in terms of osteoporosis. On the other hand, in postmenopausal women, the percentage of osteoporosis was similar, 14.7% in the control and 19.3% in the experimental group, which is in accordance with previous research [56].

Nevertheless, our results confirm that postmenopausal women with RA had the most prominent reduction in BMD. Higher percentage of osteopenia and osteoporosis was observed in PostM RA patients compared to their healthy controls and PreM RA, which can indicate that menopause and RA together amplify decrease in BMD, especially in terms of osteopenia. These findings are in line with previous results [54]. In PostM women, bone homeostasis is impaired by aging and deficiency in estrogens. In addition to their role in maintaining bone integrity, estrogen hormones are involved in inflammation. The decreased estrogen concentration leads to dysregulation of the immune system, which is manifested by an asymmetric distribution of T-helper 17 (Th17) cells. This change leads to a shift towards an elevation in inflammatory cytokines, especially TNF*α*, IL-17, and RANKL, with accelerated bone loss [55]. Moreover, proinflammatory cytokines IL-1, IL-6, and TNF*α* increase after natural menopause and decrease after estrogen therapy [57]. In addition to estrogen deficiency, PostM women with osteoporosis exhibit an increased oxidative stress associated with a decreased antioxidant defence compared to the healthy population [58].

In spite of the treatment protocol with methotrexate and low dose of prednisolone, only 20% of patients were in remission of the disease. This has been confirmed by the persistently increased CRP and ESR levels in the patient group and DAS28 value. Despite the fact that most prevalent comorbidities in the patient group were hypertension and arrhythmia, patients did not suffer from previous CVD events. Previous studies [59, 60] showed that ACE inhibitors and beta-blockers, as most frequently used drugs in our sample of population, could influence oxidative stress. We cannot take such an attitude and make a positive correlation between the reduction or elevation of oxidative stress and the intake of these drugs on redox status, since we have almost similar number of patients treated with these drugs in the both groups. Taking into account all abovementioned, we can assume that redox alterations in these patients were not influenced by their chronic therapy, since we had similar number of treated participants, both in control and RA groups.

Our data suggest an important role of oxidative stress and postmenopausal estrogen deficiency in decreasing bone density and enhancing inflammation in RA women. Therefore, anti-inflammatory therapy may be supplemented with therapeutic approaches such as estrogens, antioxidants, and physical activity [61, 62].

## 5. Conclusion

In conclusion, shifting the redox status towards increased prooxidative parameters with decreased antioxidative protection leads to oxidative stress, which can contribute to tissue and cartilage damage in both premenopausal and postmenopausal women with diagnosed RA. Our data indicate that excessive free radical production rather than impaired antioxidant defence is the main cause of oxidative stress in RA, regardless of age. Given that postmenopausal state was associated with elevated oxidative stress within healthy women and that menopausal state did not affect redox homeostasis within RA patients, but the redox homeostasis was altered in both RA groups compared to healthy women, it can be presumed that impaired redox status in RA occurred due to presence of the disease, irrespective of age. Menopause had the biggest impact on index of lipid peroxidation in healthy women, without significant influence on difference within RA patients. This can lead to the presumption that disease has the highest influence on redox status alteration, whereby influence of menopause and hormonal changes ceased to exist in RA patient's due to existence of the disease. Premenopausal women with RA are in the same risk of redox alteration as well as women with established disease in menopause. Moreover, postmenopausal women with RA had the most prominent reduction in BMD. These results may indicate the need for therapeutic use of antioxidants in the form of supplements in women with RA, regardless of age.

## Figures and Tables

**Table 1:** Laboratory parameters in the study population.

Subjects' characteristics	Healthy controls		Rheumatoid patients	
	Premenopausal control (1)	Postmenopausal control (2)	Premenopausal patient (3)	Postmenopausal patient (4)
ESR (mm/h)	11.2 ± 10.3^b^^∗∗^^,c^^∗∗^	14.2 ± 8.1^d^^∗^^,e^^∗∗^	25.3 ± 15.1	22.1 ± 13.1
CRP (mg/L)	1.8 ± 1.1^b^^∗∗^^,c^^∗∗^	2.8 ± 2.7^d^^∗∗^^,e^^∗^	11.6 ± 17.2	6.7 ± 9.2
Fibrinogen (g/L)	3.1 ± 0.6^b^^∗∗^	3.3 ± 0.5^d^^∗^	3.8 ± 0.6	3.5 ± 0.7
Cholesterol (mmol/L)	5.8 ± 1.0^a^^∗∗^	6.8 ± 1.2^d^^∗∗^	5.5 ± 0.9^f^^∗^	6.3 ± 1
Triglycerides (mmol/L)	1.4 ± 0.4	1.4 (0.7–3.9)	1.4 (0.7–3.3)	1.6 ± 0.7
HDL (mmol/L)	1.6 ± 0.3	1.6 (1.2–2.5)^d^^∗^	1.3 (1.1–2.0)	1.5 ± 0.3
LDL (mmol/L)	3.5 ± 0.9^a^^∗∗^	4.5 ± 1.1^d^^∗∗^^,e^^∗^	3.5 ± 0.8	3.9 ± 1

The values are expressed as means ± SD or median (range). ESR: erythrocyte sedimentation rate; CRP: C-reactive protein; HDL: high-density lipoprotein; LDL: low-density lipoprotein; ^∗^statistical significance (*p* < 0.05); ^∗∗^statistical significance (*p* < 0.01). Statistical significance between groups are as follows: ^a^1 and 2; ^b^1 and 3;^c^1 and 4; ^d^2 and 3; ^e^2 and 4; ^f^3 and 4. Detailed laboratory parameters and clinical characteristics of the study population in the followed subgroups are presented in our previous work [11].

**Table 2:** Comorbidities in the study population.

Comorbidities	Healthy controls		Rheumatoid patients	
	Premenopausal control (*n* = 14)	Postmenopausal control (*n* = 34)	Premenopausal patient (*n* = 11)	Postmenopausal patient (*n* = 31)
Hypertension	2 (14.9)	16 (47.1)	1 (9.1)	17 (54.8)
Arrhythmia	2 (14.9)	2 (5.9)	2 (18.2)	3 (9.7)
Hyperthyroidism	1 (2.8)	1 (2.9)	1 (9.1)	/
Hashimoto thyroiditis	1 (2.8)	1 (2.9)	1 (9.1)	2 (6.5)
Hypercholesterolemia	/	2 (5.9)	1 (9.1)	1 (3.2)
Gallbladder calculosis	/	/	/	2 (6.5)
Bronchial asthma	/	/	/	2 (6.5)
Peptic ulcer	1 (2.8)	2 (5.9)	/	3 (9.7)
Gout	/	1 (2.9)	/	/
Glaucoma	1 (2.8)	/	/	/
Migraine	/	1 (2.9)	/	/

Data are presented as a number of participants (%).

**Table 3:** Drugs used for chronic diseases treatment in the study population.

Chronic therapy		Healthy controls		Rheumatoid patients	
		PreM control (*n* = 14)	PostM control (*n* = 34)	PreM patient (*n* = 11)	PostM patient (*n* = 31)
ACE inhibitors	Captopril	4 (28.6)	14 (41.2)	3 (27.3)	11 (35.5)
	Enalapril				
	Ramipril				
	Perindopril				
	Trandolapril				
	Cilazapril				
	Perindopril+indapamid				
	Lisinopril+hydrochlorothiazide				
	Ramipril+ hydrochlorothiazide				
	Quinapril+hydrochlorothiazide				

Beta-blockers	Atenolol	4 (28.6)	5 (14.7)	1 (9.1)	9 (29.0)
	Metoprolol				
	Bisoprolol				
	Nebivolol				
	Bisoprolol+hydrochlorothiazide				

Ca-antagonists	Amlodipine	/	2 (5.9)	1 (9.1)	2 (6.5)
	Felodipine				

Diuretics	Indapamide	/	1 (2.9)	/	/

Statins	Atorvastatin	/	2 (5.9)	1 (9.1)	1 (3.2)
	Rosuvastatin				

Phlebotonics	Diosmine	/	1 (2.9)	2 (18.2)	/

Thionamide	Propylthiouracil	1 (7.1)	1 (2.9)	1 (9.1)	/

Thyroid hormones	Levothyroxin	/	2 (5.9)	2 (18.2)	4 (12.9)

Proton pump inhibitors	Pantoprazol	1 (7.1)	2 (5.9)	2 (18.2)	6 (19.4)

Bronchodilator	Aminophyline	/	/	/	2 (6.5)
	Fenoterol+iprathropium bromide				

H_1_-antagonists	Hydroxizine hydrochloride	/	2 (5.9)	/	/
	Cinnarizine				

Xanthine oxidase inhibitor	Alopurinol	/	1 (2.9)	/	/

Prostaglandin analogues	Latanoprost	1 (7.1)	/	/	/

Ergot alkaloids	Dihydroergotamine	/	1 (2.9)	/	/

Data are presented as a number of participants using specific group of drug (%). PreM: premenopausal; PostM: postmenopausal.

**Table 4:** BMD according to DXA.

Subjects' characteristics	Healthy controls		Rheumatoid patients	
	Premenopausal control (1)	Postmenopausal control (2)	Premenopausal patient (3)	Postmenopausal patient (4)
Normal BMD	10 (71.4%)	15 (44.1%)	4 (36.4%)	3 (9.7%)
Osteopenia	4 (28.6%)	14 (41.2%)	7 (63.6%)	22 (71.0%)
Osteoporosis	/	5 (14.7%)	/	6 (19.3%)

The values are expressed as number (percent). BMD: bone mineral density.

## Data Availability

The data used to support the findings of this study are available from the corresponding author upon request.
